# ACE2-Ang-(1-7)-Mas Axis in Brain: A Potential Target for Prevention and Treatment of Ischemic Stroke

**DOI:** 10.2174/1570159X11311020007

**Published:** 2013-03

**Authors:** Teng Jiang, Li Gao, Jie Lu, Ying-Dong Zhang

**Affiliations:** a Department of Neurology, Nanjing First Hospital, Nanjing Medical University, Nanjing, P.R. China; b Department of Neurology, Nanjing Brain Hospital, Nanjing Medical University, Nanjing, P.R. China

**Keywords:** Renin-angiotensin system, Angiotensin-(1-7), Stroke, Neuroprotection, Oxidative stress.

## Abstract

The renin-angiotensin system (RAS) in brain is a crucial regulator for physiological homeostasis and diseases of cerebrovascular system, such as ischemic stroke. Overactivation of brain Angiotensin-converting enzyme (ACE) - Angiotensin II (Ang II) - Angiotensin II type 1 receptor (AT_1_R) axis was found to be involved in the progress of hypertension, atherosclerosis and thrombogenesis, which increased the susceptibility to ischemic stroke. Besides, brain Ang II levels have been revealed to be increased in ischemic tissues after stroke, and contribute to neural damage through elevating oxidative stress levels and inducing inflammatory response in the ischemic hemisphere *via* AT_1_R. In recent years, new components of RAS have been discovered, including ACE2, Angiotensin-(1–7) [Ang-(1-7)] and Mas, which constitute ACE2-Ang-(1-7)-Mas axis. ACE2 converts Ang II to Ang-(1-7), and Ang-(1-7) binds with its receptor Mas, exerting benefical effects in cerebrovascular disease. Through interacting with nitric oxide and bradykinin, Ang-(1-7) could attenuate the development of hypertension and the pathologic progress of atherosclerosis. Besides, its antithrombotic activity also prevents thrombogenic events, which may contribute to reduce the risk of ischemic stroke. In addition, after ischemia insult, ACE2-Ang-(1-7)-Mas has been shown to reduce the cerebral infarct size and improve neurological deficits through its antioxidative and anti-inflammatory effects. Taken together, activation of the ACE2-Ang-(1-7)-Mas axis may become a novel therapeutic target in prevention and treatment of ischemia stroke, which deserves further investigations.

## INTRODUCTION 

Stroke, usually caused by a temporary or permanent reduction of local cerebral blood flow, is a major cause of mortality and disability in the world [1]. Among all stroke cases, ischemic stroke accounts for approximately 70% in China [2] and 80%-85% in Western countries [3]. Several physiopathologic conditions, such as hypertension, atherosclerosis and prothrombotic state, have been found to increase the risk for ischemic stroke. In addition, elevated oxidative stress levels and inflammatory response in brain at the early stages of ischemic stroke have been also revealed to contribute to the irreversible cerebral damage after ischemia insult. On the other hand, the renin-angiotensin system (RAS) in brain acts as a crucial regulator for physiological homeostasis and diseases of the cerebrovascular system, including ischemic stroke. The deleterious effects of brain Angiotensin-converting enzyme (ACE) - Angiotensin II (Ang II) - Angiotensin II type 1 receptor (AT_1_R) axis in the pathogenesis of ischemic stroke were well elucidated by several studies. Recently, new components of RAS, such as ACE2, Angiotensin-(1–7) [Ang-(1-7)] and Mas, have been identified in brain. There is emerging evidence that the ACE2-Ang-(1-7)-Mas axis in brain exerts mainly beneficial effects against thephysiopathologic conditions related to ischemic stroke. Activation of this axis was reported to attenuate the development of hypertension [4] and the pathologic progress of atherosclerosis [5-7]. Meanwhile, its antithrombotic activity also prevents thrombogenic events, which may contribute to reduce the risk of ischemic stroke [8]. Moreover, ACE2-Ang-(1-7)-Mas axis was also found to be neuroprotective after ischemia insult, which counteracted the harmful contributions of ACE-Ang II-AT_1_R axis [9, 10]. Here, we review the protective effects of ACE2-Ang-(1-7)-Mas axis on prevention and treatment of ischemic stroke. In addition, we also discuss the underlying mechanisms referring to these beneficial effects in this article. 

## THE CLASSIC PATHWAY AND THE NEW COMPONENTS OF BRAIN RAS IN THE PATHOGENESIS OF ISCHEMIC STROKE 

Brain RAS is physically separated from the RAS in peripheral organs by the presence of the blood-brain barrier, which prevents the diffusion of Ang II from the circulation into the brain [11]. However, the RAS in brain contains the same elements as the RAS in peripheral tissues, such as ACE, Ang II and AT_1_R, which makes up the classic pathway of RAS. The classic pathway involves a two-step enzymatic pathway. First, the aspartyl protease renin, which is primarily released by the kidneys, cleaves a hepatic protein, angiotensinogen, to angiotensin I (Ang I). The second step involves hydrolysis of Ang I by ACE, resulting in the production of the bioactive octapeptide Ang II [12]. There is increasing evidence that the classic pathway of brain RAS has been implicated in the pathogenesis and outcome of ischemic stroke. Overactivation of brain ACE-Ang II-AT_1_R axis was found to take part in the development and maintenance of hypertension, an important risk factor for ischemic stroke, by elevating oxidative stress in brain and increasing the activity of sympathetic nervous system [13]. Hypertension is often associated with prothrombotic state, which has been considered as another risk factor of ischemic stroke. An increased number of studies indicated that Ang II promoted thrombosis *in vivo*, by interacting with AT_1_R [14, 15]. Ang II also participated in the progress of atherosclerosis through evoking vascular smooth muscle cell (VSMC) growth and migration [16] and inducing endothelial dysfunction [17], thus increasing the risk of ischemic stroke. Besides, Ang II levels have been found to be temporarily increased after ischemic stroke in rat brain [18], and contribute to neural damage through elevating oxidative stress levels, inducing inflammatory response and reducing cerebral perfusion in the ischemic hemisphere *via *AT_1_R [19]. 

In addition to the classic pathway, the other components of RAS including ACE2, Ang-(1-7) and Mas, have been also identified in the central nervous system (CNS). As the first known human homologue of ACE [20], ACE2 is widespread throughout the rodent brain, including neurons [21], astrocytes [22], and endothelial and smooth muscle cells of cerebral arteries [23]. Functionally, ACE2 acts as a carboxypeptidase to cleave the COOH-terminal leucyl residue from Ang I, thus producing Ang-(1-9). More importantly, the enzyme is also able to hydrolyze Ang II to produce Ang-(1-7), at a much higher efficiency than that for Ang I to Ang-(1-9) [24]. Similar as ACE2, expression of Mas was observed in neurons [25], astrocytes [26] and endothelial cells of cerebral resistance vessels [27]. In 1988, Young *et al*. first observed the presence of Mas in the hippocampus and cortex of rat brain [28], later studies extended the observation to other brain structures, especially the cardiovascular regulatory centers in the brain, such as nucleus tractus solitarii (NTS), rostral ventrolateral medulla (RVLM) and paraventricular nucleus (PVN) [29]. As the ligand for the Mas receptor [30], Ang-(1-7) is also present as an endogenous constituent of the brain, in areas including the hypothalamus, medulla oblongata, and amygdale [31]. In brain, Ang-(1-7) is synthesized predominately from degrading Ang II by ACE2 [32] whilst a few researches revealed that Ang-(1-7) can be also directly formed from Ang I by the action of neutral endopeptidase (also known as neprilysin) and prolyl-endopeptidase [33]. After synthesis, Ang-(1-7) can be cleaved into Ang-(1-5) or Ang-(1-4) by ACE [34] or neprilysin [35], respectively. Together, the three components make up ACE2-Ang-(1-7)-Mas axis, the new arm of the RAS in brain. Emerging evidence suggested that activation of ACE2-Ang-(1-7)-Mas axis could attenuate the development of hypertension and the pathologic progress of atherosclerosis. Its antithrombotic activity also prevented thrombogenic events, which may reduce the susceptibility to ischemic stroke. Besides, ACE2-Ang-(1-7)-Mas has been shown to protect against ischemic damage through its antioxidative and anti-inflammatory effects after ischemia insult.

## ANTIHYPERTENSIVE EFFECT OF BRAIN ACE2-ANG-(1-7)-MAS AXIS AND RELATED MECHANISMS 

As the most important modifiable risk factor for stroke [36], hypertension is involved in the pathogenesis of ischemic stroke through accelerating the progression of atherosclerosis, which leads to the thrombogenesis in cerebral circulation [37]. It is widely accepeted that antihypertensive therapy is important for prevention of stroke, regardless of age, gender, or ethnicity [38]. Recently, several studies revealed that the brain ACE2-Ang-(1-7)-Mas axis acted as a pivotal regulator of blood pressure (BP), which counteracted the pressor effect of ACE-Ang II-AT_1_R in brain [4].

In 2008, Diz *et al*. injected the selective ACE2 inhibitor MLN4760 into the NTS of Sprague-Dawley (SD) rats and observed a long-lasting reduction in mean arterial pressure (MAP) [39]. Yamazato *et al*. found that lentiviral-mediated overexpression of ACE2 in RVLM of spontaneously hypertensive rat (SHR) caused a significantly reduction in MAP [40]. In a recent study from Xia *et al*., overexpression of ACE2 in the brain was observed to prevent the development of hypertension in a triple transgenic mouse model, which was generated by transposing the hACE2 phenotype onto the R^+^A^+^ chronically hypertensive mouse background [41]. More recently, Feng *et al*. revealed that overexpression of ACE2 in mice brain attenuated the development of neurogenic hypertension caused by Ang II [42]. Meanwhile, bilateral microinjection of an adenovirus encoding hACE2 into the PVN of SD rats was found to markedly blunt the hypertension induced by Ang II [43]. Similar to ACE2, microinjection of Ang-(1-7) into the NTS was found to induce significant reductions in MAP in both Wistar rats and SHR [44]. A study from Höcht *et al*. showed that intrahypothalamic administration of Ang-(1-7) not only caused a significant reduction in MAP, but also abolished the pressor response induced by Ang II in sinoaortic denervated rats [45]. In agreement with that result, Cerrato *et al*. revealed that injection of Ang-(1-7) into the anterior hypothalamic area markedly decreased MAP in SHRs [46]. In 2003, Dobruch *et al*. demonstrated that intracerebroventricular (I.C.V) infusion of Ang-(1-7) led to a significant decrease in BP in TGRmRen2 (27) rats with renin dependent transgenic hypertension [47]. Similar result was obtained by Guimaraes *et al*. that I.C.V infusion of Ang-(1-7) for 4 weeks attenuated the increase in MAP in a rat hypertension model induced by deoxycorticosterone acetate-salt (DOCA) [48]. 

The role of brain ACE-Ang II-AT_1_R axis in the pathogenesis of hypertension has been well elucidated by previous studies [13]. In the past years, many studies have demonstrated that ACE2-Ang-(1-7)-Mas axis may exert its antihypertensive effect by directly affecting the classic components of the RAS in CNS. In an *in vitro* study by Xiao *et al*., overexpression of ACE2 in catecholaminergic neurons was found to blunt the Ang II-induced upregulation of AT_1_R [49]. *In vivo*, Xia *et al*. found that overexpression of ACE2 in the brain reduced local Ang II level in a triple transgenic mouse model while Feng *et al*. revealed that overexpression of ACE2 in the subfornical organ of mouse brain caused a reduction in AT_1_R expression [50]. Recently, these authors extended their findings by showing that angiotensin II type 2 receptor (AT_2_R) to AT_1_R and Mas to AT_1_R ratios were significantly increased in hACE2 transgenic mice [42]. These data were confirmed by a study from Sriramula *et al*. that ACE2 overexpression significantly decreased AT_1_R and ACE expression and increased AT_2_R and Mas expression in the PVN of male SD rat [43]. Our group also showed that I.C.V infusion of Ang-(1-7) for 4 weeks downregulated the expression of AT_1_R which was accompanied by a reduction in Ang II levels in SHR brain (unpublished data).

Baroreflex is one of the most important physiological mechanisms involved in BP regulation, which became less sensitive in hypertensive individual due to changes in vascular distensibility and altered activity in the brainstem portion of the reflex [51]. Emerging data indicated that restoration of impaired baroreflex function represent one mechanism that contributed to the antihypertensive actions of brain ACE2-Ang-(1-7)-Mas axis. ACE2 overexpression in brain normalized the baroreflex function impairment, enhanced parasympathetic tone, and reduced sympathetic activity in chronically hypertensive mice [41]. It also significantly inhibited the decrease in baroreflex sensitivity and parasympathetic activity in a mice hypertension model induced by low-dose Ang II infusion [42]. Besides, Xiao *et al*. showed that global overexpression of exogenous ACE2 in the brain prevented the impairment in baroreflex sensitivity and decreased sympathetic nerve activity in the congestive heart failure (CHF) mice [52]. In line with ACE2, I.C.V infusion of Ang-(1-7) caused a significantly increase in baroreflex sensitivity in male Wistar rats [53], and this effect was amplified by co-infusion with bradykinin, suggesting a potential synergistic effect between the two peptides [54]. I.C.V infusion of Ang-(1-7) was also found to enhanced baroreflex function in rabbits with CHF through inhibiting sympathetic outflow and increasing vagal outflow [55]. In addition, infusion Ang-(1-7) into lateral cerebral ventricle of DOCA rats was found to cause an improvement of baroreflex function and a restoration of the sympathetic nerve activity [48].

As a highly reactive, diffusible, and unstable radical, nitric oxide (NO) in the CNS was found to act as a vasodilatory and sympatho-inhibitory molecule, and participate in central mechanisms of BP regulation [56]. In 1993, Calka and Block first observed the codistribution of Ang-(1-7) with NO synthase in neurons of PVN, suggesting a potential link between this heptapeptide and NO [57]. In human endothelial cells, Ang-(1-7) led to long-lasting endothelial NO synthase (eNOS) phosphorylation and stimulated NO release *via *PI3K-Akt-PKB pathway [58]. *In vivo*, a study from Feng and colleagues revealed that ACE2 overexpression resulted in increased endothelial and neuronal NO synthase (nNOS) and NO levels in the brain, and prevented the Ang II-mediated decrease in NO synthase expression in BP regulatory centers in the brain of hACE2 transgenic mice [42]. In a more recent study from Cerrato *et al*., infusion of Ang-(1-7) into the anterior hypothalamic area of SHR markedly upregulated nNOS expression, which may serve as a compensatory and protective mechanism to combat hypertension [46].

Elevated oxidative stress levels and increased expression of proinflammatory cytokines in brain were revealed to participate in the development and maintenance of hypertension through activating redox signaling in the BP regulatory centers [59, 60]. An *in vitro* study from Xia *et al*. revealed that ACE2 overexpression was related to a reduction of reactive oxygen species (ROS) formation in a mouse neuroblastoma cell line treated with Ang II [61]. *In vivo*, they also found that PVN-targeted ACE2 over-expression significantly decreased Ang II-induced ROS formation *via *NADPH oxidase inhibition and attenuated the increase in the expression of tumour necrosis factor (TNF)-α, interleukin (IL)-1β and IL-6 in brain of ACE2 knockout mice [43]. Consistent with these findings, a recent study from our group demonstrated that I.C.V infusion of Ang-(1-7) for 4 weeks markedly reduced the levels of malondialdehyde (MDA) and gp91^phox^, a subunit of NADPH, which was associated with an increase in superoxide dismutase (SOD) activity in brain of SHR (unpublished data). These findings indicated that the inhibition of oxidative stress and inflammation may represent part of the underlying mechanisms for the antihypertensive effects of ACE2-Ang-(1-7)-Mas axis. 

Additionally, Ang-(1-7) was found to inhibit neuronal activity in BP regulatory centers [62] through preventing norepinephrine release [63], inhibiting activity and expression of tyrosine hydroxylase [64] and activating hyperpolarizing I_Kv_ in catecholaminergic neurons in a NO-dependent manner [65], which may also contribute to its antihypertensive effect.

## ANTI-ATHEROSCLEROTIC AND ANTITHROMBOTIC ACTIONS OF ACE2-ANG-(1-7)-MAS AXIS 

Atherosclerosis represents the most common cause of ischemic stroke [66]. It is now widely agreed that endothelial dysfunction and proliferation of VSMC are involved in the development of atherosclerotic plaques, and rupture of atherosclerotic plaque usually triggers plaque thrombosis, blocking the cerebral arteries and leading to the ischemic stroke [67].

In the recent years, several studies have revealed that activation of ACE2-Ang-(1-7)-Mas axis was able to attenuate the progress of atherosclerosis through inhibiting VSMC proliferation and restoring endothelial function, which may reduce the risk of ischemic stroke. Lovren’s study in apolipopretein E (ApoE)-knockout mice revealed the improvement of endothelial dysfunction in an Ang-(1-7)-dependent manner by the overespression of ACE2 [68]. In a relatively large cohort animal study from Zhang *et al*., aortic segments from rabbits transfected by Ad-ACE2 showed significantly inhibited fatty streak formation, neointimal macrophage infiltration, and alleviation of impaired endothelial function, associated with decreased expression of monocyte chemoattractant protein-1 (MCP-1), lectin-like oxidized low-density lipoprotein receptor-1 (LOX-1), and proliferating cell nuclear antigen (PCNA), which delayed the onset of atherosclerotic lesions [5]. Moreover, Dong *et al*. observed that overexpression of ACE2 resulted in stable plaque compositions, such as fewer macrophages, less lipid deposition and more collagen contents in the abdominal aorta of rabbits [69]. Just like ACE2, chronic Ang (1-7) treatment was revealed to significantly improve endothelial function and inhibit atherosclerotic lesion through AT_2_R and Mas receptor in vessels of ApoE - deficient mice, which was associated with decreased superoxide production and increased endothelial nitric oxide synthase immunoreactivity [6]. Moreover, Ang-(1-7) was found to inhibit Ang II-induced VSMC proliferation and migration, partially through negative modulating Ang II induced ERK1/2 activity [70]. On the other hand, many studies revealed that AVE 0991 as a non-peptide Mas agonist could ameliorate atherosclerosis progression [7], which was associated with a significant reduction of NADPH oxidase expression in ApoE - knockout mice [71]. More recently, Sheng-Long *et al*. reported that AVE0991 was able to attenuate Ang II-induced VSMC proliferation in a dose-dependent fashion through modulating Mas/HO-1/p38 MAPK related signaling pathway [72].

Additionally, activation of ACE2-Ang-(1-7)-Mas axis was shown to produce antithrombotic activity by recent studies, which may contributed to prevent thrombogenic events, such as ischemic stroke. Fraga-Silva *et al*. reported that activation of ACE2 significantly inhibited thrombosis and reduced platelet attachment to vessels in SHRs [8]. In another study from Fraga-Silva *et al*., Ang-(1-7) was found to inhibit thrombus formation in Mas^+/+^ mice, and this effect was abolished in Mas-knockout mice. Besides, they also revealed that Ang-(1-7) released NO from rat and Mas^+/+^ mouse platelets, which was blocked by A-779. These findings indicated that Mas-mediated NO release from platelets was contributed to the antithrombotic effect of Ang-(1-7) [73]. Moreover, Kucharewicz and colleagues reported that intravenous infusion of Ang-(1-7) into rats caused 50% to 70% reduction of the thrombus weight, which was dose-dependently reversed by co-treatment with A-779, NO synthase inhibitor or prostacyclin synthesis inhibitor. They also observed that the antithrombotic effects of captopril and losartan were attenuated by A-779 in a dose-dependent manner, indicating the antithrombotic effect of angiotensin converting enzyme inhibitors and angiotensin receptor blockers were partially mediated by Ang-(1-7)-evoked release of NO and prostacyclin [74].

## NEUROPROTECTIVE EFFECT OF BRAIN ACE2-ANG-(1-7)-MAS AXIS AFTER ISCHEMIA INSULT AND RELATED MECHANISMS 

The neuroprotection of ACE2-Ang-(1-7)-Mas axis after ischemia insult has been verified in two different rat models of cerebral ischemia. In rats with endothelin (ET)-1 induced transient middle cerebral artery occlusion (tMCAO), I.C.V infusion of Ang-(1-7) or diminazine aceturate, an ACE2 activator, could significantly attenuate the cerebral infarct size and neurological deficits after the ischemia insult [9]. Meanwhile, in a recent study from our group, central administration of Ang-(1-7) was found to minimize the size of cerebral infarction and improved neurological functions in a rat model of permanent middle cerebral artery occlusion (pMCAO) [10]. These beneficial actions were fully reversed by co-infusion of A-779, indicating the neuroprotective effect of ACE2 and Ang-(1-7) was mediated by Mas receptor.

Oxidative stress has a detrimental effect in the progress of ischemic stroke, since the brain has large amounts of polyunsaturated fatty acids, thus particularly vulnerable to oxygen free radical attack [75]. MDA is a biomarker of oxidative stress and rapidly elevated after ischemic stroke subjects [76], positively correlating with infarct size, stroke severity and patient outcome. SOD represents another potential biomarker of oxidative stress in stroke. As the first line of defense against oxidative stress, it catalyzed the dismutation reaction of superoxide anion to the more stable hydrogen peroxide [77]. I.C.V infusion of Ang-(1-7) has been found to markedly attenuate the ischemia-induced increase in MDA levels. It also enhanced total SOD activity in peri-infarct tissues of rat brain. These effects were abolished by A-779, suggested an involvement of Mas receptor in the antioxidative action of Ang-(1-7) [10].

Inflammatory response also played a crucial role in the pathophysiology of ischemia stroke [78]. Nuclear Factor-κB (NF-κB), a well-characterized transcriptional regulator involved in neuroinflammation, is activated after cerebral ischemia and contributes to infarction [79]. Meanwhile, as target genes of NF-κB, several pro-inflammatory cytokines and enzymes, such as TNF-α, IL-1β and cyclooxygenase (COX)-2 [80-82] are upregulated and lead to neuronal damage after cerebral ischemia. In a recent study from our group, I.C.V infusion of Ang-(1-7) was found to cause a significant reduction in NF-κB activity, which was associated with decreased expression of TNF-α, IL-1β and COX-2 in peri-infarct tissues of rat brain, indicating that suppressing inflammation at initial phase of cerebral ischemia by inhibiting NF-κB activity has contributed to the neuroprotection of Ang-(1-7) [10].

The role of NO in the pathogenesis of ischemic stroke was double-sided and somewhat ambiguous. Formation of nitric oxide by eNOS immediately after cerebral ischemia has been proven to be protective, since it promoted collateral circulation and restored cerebral blood flow during the early stages of cerebral ischemia [83, 84]. However, with prolongation of ischemia or during subsequent reperfusion, the formation of nitric oxide by inducible NO synthase (iNOS) in activated microglia was reported to be deleterious, which took part in the irreversible ischemic brain injury [85, 86]. Our group revealed that I.C.V infusion of Ang-(1-7) markedly enhanced NO levels during the early stages of cerebral ischemia. In addition, it also significantly stimulated eNOS mRNA and protein expression within 48 hours of the onset of pMCAO [87]. Meanwhile, in a recent study from Mecca *et al*., I.C.V administration of Ang-(1-7) significantly attenuated the ischemia-induced increase of iNOS mRNA expression in the ischemic tissues following ET-1-induced tMCAO [9]. Hence, the different ways in the regulation of NO synthase may represent part of the underlying mechanisms for the neuroprotective effect of Ang-(1-7).

The neuroprotective effect of bradykinin (BK) *via *the kinin B2 receptor in ischemic stroke has been revealed by several studies [88, 89]. Tom *et al*. revealed that Ang-(1-7) was able to inhibit the proteolytic function of ACE by binding with ACE at the COOH-terminal domain, thus promoting bradykinin function [90]. Moreover, in a previous study from our group, I.C.V infusion of Ang-(1-7) markedly enhanced BK levels from 6 h to 48 h in ischemic brain tissues after reperfusion [91]. These findings suggested that the neuroprotective effect of Ang-(1-7) may be partially mediated by the interaction between this heptapeptide and BK.

## CONCLUSIONS AND PERSPECTIVES 

**Fig. (1):**
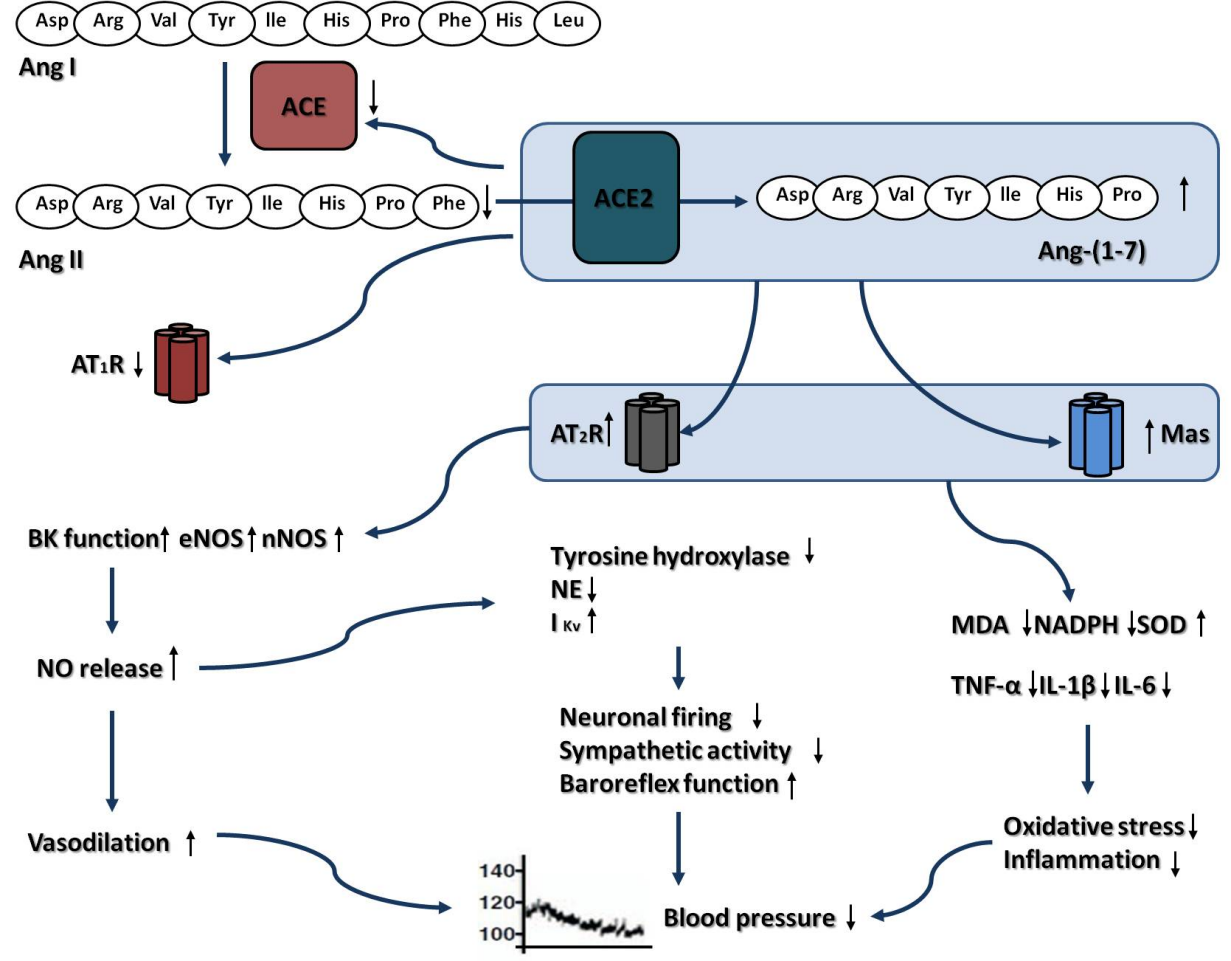
Depressor effect of brain ACE2-Ang-(1-7)-Mas axis and the related mechanisms. Abbreviations: ACE, Angiotensin converting enzyme; Ang I, Angiotensin I; Ang II, Angiotensin II; Ang-(1-7), Angiotensin-(1-7); AT_1_R , Angiotensin II type 1 receptor; AT_2_R , Angiotensin II type 2 receptor; BK, Bradykinin; eNOS, Endothelial nitric oxide synthase; I_Kv,_ Delayed rectifier K^+^ current; IL-1β, Interleukin-1β; IL-6, Interleukin-6; MDA, Malondialdehyde; NE, Norepinephrine; nNOS, Neuronal nitric oxide synthase; NO, Nitric oxide; SOD, Super oxygen dehydrogenises; TNF-α, Tumour necrosis factor–α.

**Fig. (2):**
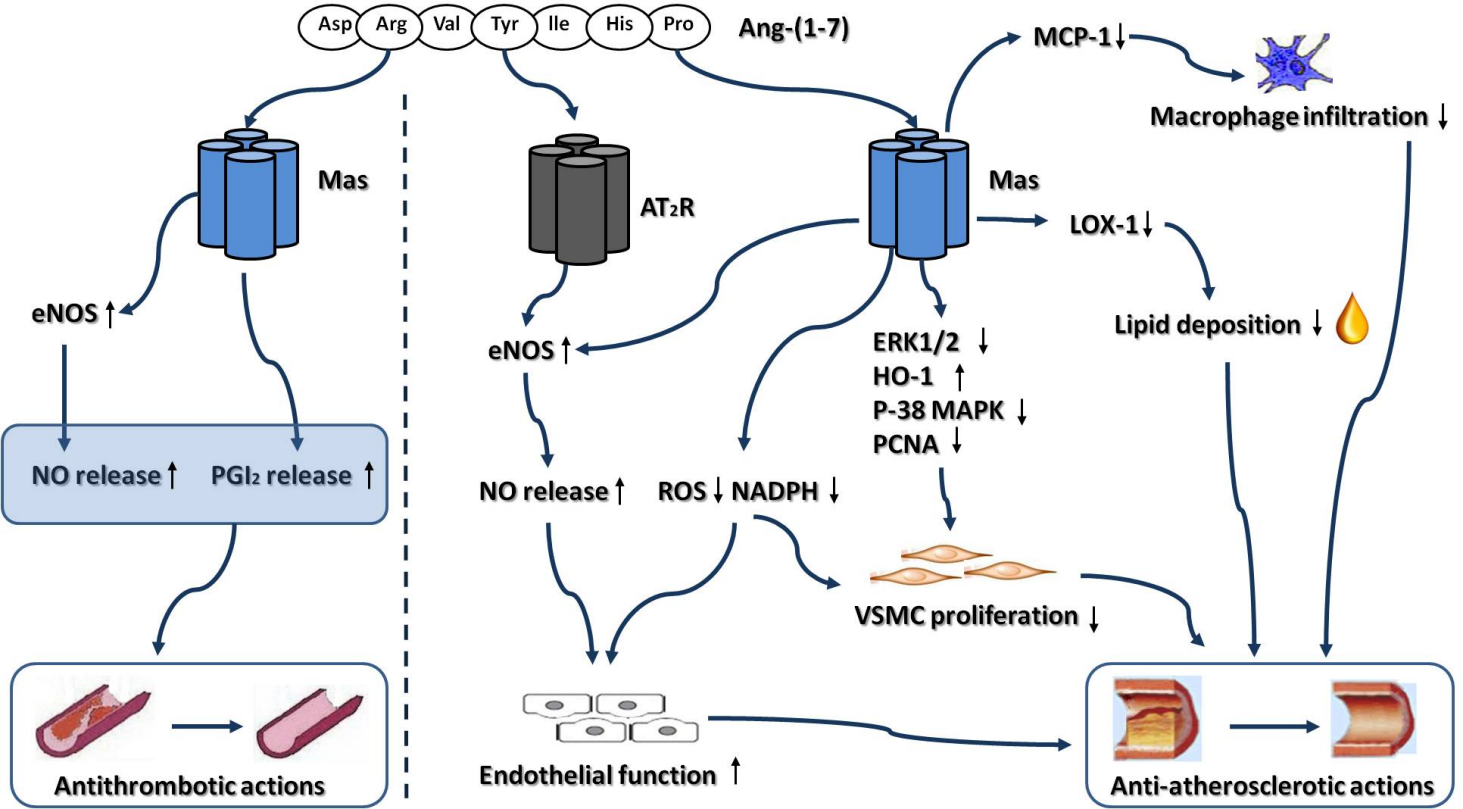
Anti-atherosclerotic and antithrombotic actions of ACE2-Ang-(1-7)-Mas axis. Abbreviations: Ang-(1-7), Angiotensin-(1-7); AT_2_R , Angiotensin II type 2 receptor; BK, Bradykinin; eNOS, Endothelial nitric oxide synthase; ERK, Extracellular signal-regulated kinase; HO-1, Heme oxygenase-1; LOX-1, Lectin-like oxidized low-density lipoprotein receptor-1; MCP-1, Monocyte chemoattractant protein-1; NO, Nitric oxide; P38 MAPK, P38 mitogen-activated protein kinase; PCNA, Proliferating cell nuclear antigen; PGI_2_, Prostaglandin I_2_; ROS, Reactive oxygen species; VSMC, Vascular smooth muscle cell.

**Fig. (3):**
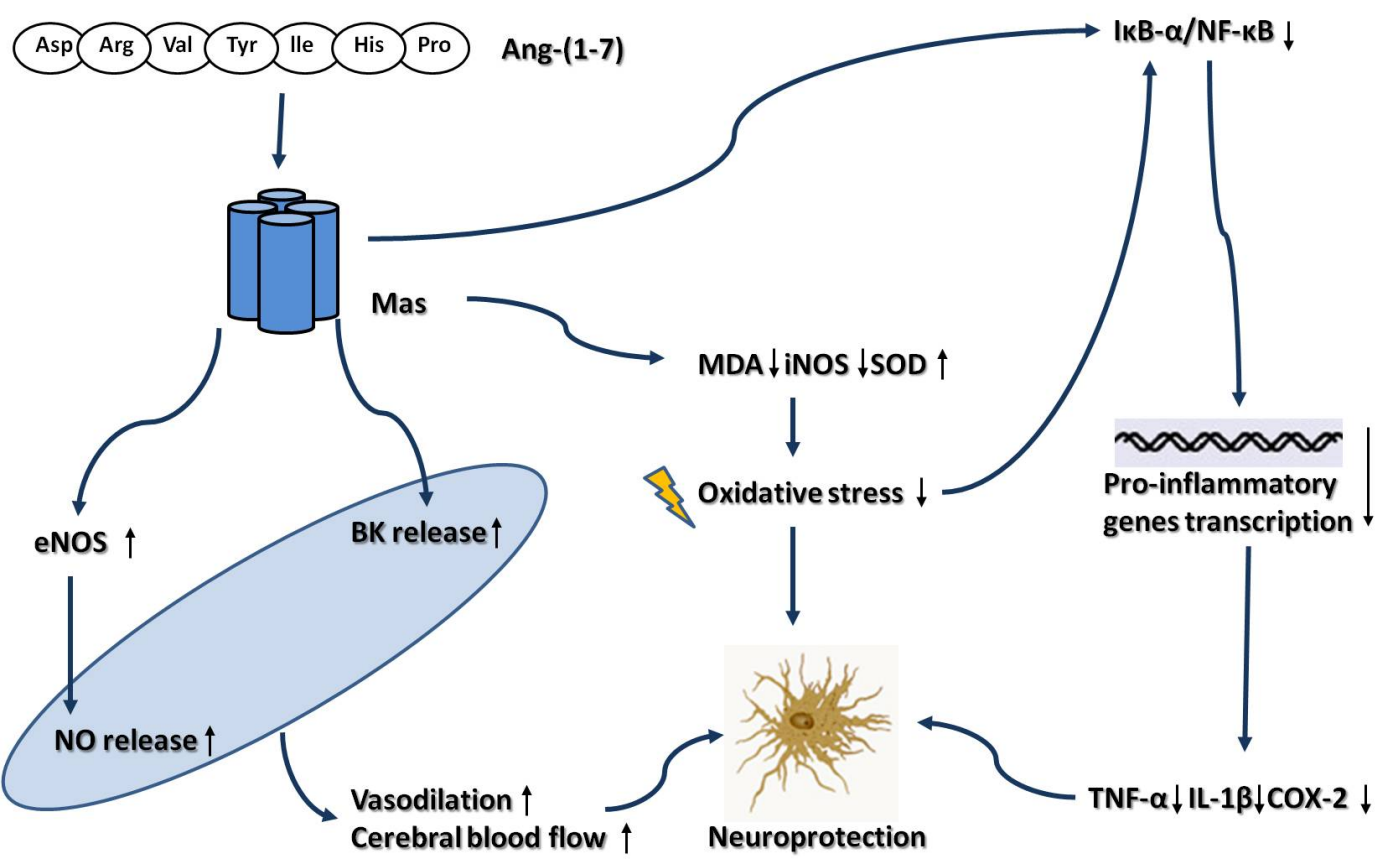
Neuroprotective effect of brain ACE2-Ang-(1-7)-Mas axis after ischemia insult. Abbreviations: Ang-(1-7), Angiotensin-(1-7); BK, Bradykinin; COX-2, Cyclooxygenase-2; eNOS, Endothelial nitric oxide synthase; IL-1β, Interleukin-1β; iNOS, Inducible nitric oxide synthase; MDA, Malondialdehyde; NF-κB, Nuclear actor-κB. NO, Nitric oxide; SOD, Super oxygen dehydrogenises; TNF-α, Tumour ecrosis factor–α.

In this review, we provide evidences from *in vitro* and *in vivo* experiments that activation of ACE2-Ang-(1-7)-Mas axis in the CNS may protect against the development of hypertension (Fig. **1**), atherosclerosis and thrombogenesis (Fig. **2**), which contribute to decrease the risk for ischemic stroke. Besides, our review of the literatures also show that the brain ACE2-Ang-(1-7)-Mas axis exerts neuroprotective effects against cerebral ischemic damage after ischemia insult, which counteracts the deleterious actions of ACE-Ang II-AT_1_R axis in brain (Fig. **3**). However, there are still some limitations which restrict the application of ACE2 and Ang-(1-7) in animal experiments. In most animal studies, ACE2 gene was transferred into brain by lentivirus. Hence, researchers did not observe the long-term protective effect of ACE2 in brain due to the limited life of the virus. Additionally, as the main factor of ACE2-Ang-(1-7)-Mas axis, Ang-(1-7) has a relatively short duration of biological effect, since it was rapidly inactivated by ACE *in vivo*. Besides, Ang-(1-7) cannot be administrated orally, due to its high water-solubility and its susceptibility to protease degradation [92]. Recently, by using genetic approaches, several transgenic mouse models was generated, which was characterized by high ACE2 expression and activity, restricted to the CNS. These transgenic animal models enabled us to investigate the beneficial of ACE2 in CNS during a long period of time [93]. Meanwhile, Marques and colleagues recently demonstrated that the inclusion of Ang-(1-7) into the oligosaccharide hydroxypropyl β-cyclodextrin cavity could protect this peptide during the passage through the gastrointestinal tract when orally administrated, which overcame the shortcomings of Ang-(1-7) [94, 95]. On the other hand, the development of AVE 0991 represented an important step for expanding the application range of Ang-(1-7). AVE 0991 is a non-peptide and an orally active Ang-(1–7) receptor Mas agonist that mimics the Ang-(1–7) effects in several organs such as kidney and heart, and directly activating Mas-dependent signalings by this compound may provide new therapeutic opportunities in the field of cerebrovascular diseases [96]. In conclusion, activation of ACE2-Ang-(1-7)-Mas axis plays a protective role in CNS. As new pharmacological and genetic approaches are becoming available, it may become an attractive target for the prevention and treatment of ischemic stroke, as well as other cerebrovascular diseases.
